# The Impact of ABO Blood Type on Developing Venous Thromboembolism in Cancer Patients: Systematic Review and Meta-Analysis

**DOI:** 10.3390/jcm10163692

**Published:** 2021-08-20

**Authors:** Fumihiko Urabe, Shoji Kimura, Kosuke Iwatani, Keiji Yasue, Yuhei Koike, Kojiro Tashiro, Shunsuke Tsuzuki, Hiroshi Sasaki, Takahiro Kimura, Shin Egawa

**Affiliations:** Department of Urology, The Jikei University School of Medicine, Tokyo 105-8461, Japan; shoji.kimura-1228@hotmail.co.jp (S.K.); ne.jp.ne.jp.ne.jp.ne.jp@gmail.com (K.I.); okei.yasue@gmail.com (K.Y.); koike.you0930@gmail.com (Y.K.); tashikoji@gmail.com (K.T.); tsuzushun60@gmail.com (S.T.); shiroshi427@gmail.com (H.S.); tkimura0809@gmail.com (T.K.); s-egpro@jikei.ac.jp (S.E.)

**Keywords:** ABO blood type, venous thromboembolism, meta-analysis, urological cancer

## Abstract

The impact of ABO blood type in the development of venous thromboembolism in cancer patients remains controversial. To develop a sense of the current opinion in this area, we conducted a systematic review and meta-analysis. In March 2021, we performed a systematic search of PubMed, the Cochrane library, and Scopus for studies that compared cancer patients who had a blood type of either O or non-O (A, B, and AB). Our objective was to use multivariate logistic regression analysis to determine how ABO blood type was associated with the development of venous thromboembolism. Our selection criteria were met by a total of nine studies in 25,884 patients for the systematic review and five studies in 22,777 patients for the meta-analysis. In cancer patients, we found that non-O blood type was associated with a nearly two-fold increase in risk of venous thromboembolism (pooled OR: 1.74, 95% CI: 1.44–2.10). Additionally, among the eligible patients, 21,889 patients were post-operative urological cancer patients. In these patients, the analysis also showed an association between non-O blood type and increasing risk of venous thromboembolism after pelvic surgery for malignancy (pooled OR: 1.73, 95% CI: 1.36–2.20). Our meta-analysis suggested that non-O blood type is a risk factor for venous thromboembolism among patients with cancer. As blood type is routinely determined preoperatively by objective and standardized methods, we anticipate that our results will be useful for managing venous thromboembolism in cancer patients, especially after pelvic surgery for urological cancers.

## 1. Introduction

Venous thromboembolism, which includes both deep venous thrombosis and pulmonary embolism, is a major cause of morbidity and mortality among patients with cancer [1,2]. In particular, venous thromboembolism is a serious and frequent complication of pelvic surgery for malignancy and is the most common cause of mortality in patients who die within 30 days after surgery [3,4].

Although our references reported several types of genetic predisposition to venous thromboembolism, such as single-nucleotide polymorphisms (SNPs), most of those variations were encountered so rarely that their clinical importance remains controversial [5]. It is thus important to characterize predisposing factors for venous thromboembolism, so that risk can be stratified accurately and prophylactic treatment strategies can be implemented effectively.

Previous hematologic studies have reported that ABO blood type is a significant genetic risk factor for venous thromboembolism [6,7]. Specifically, among the four blood groups (A, B, AB, and O), the risk of venous thromboembolism has been found to be significantly higher for non-O blood types than for type O [6,7]. However, no systematic assessment has been conducted of the relationship between blood type and venous thromboembolism risk in patients with cancer.

In this systematic review and meta-analysis, we assessed the current thinking on the impact of ABO blood type on the development of thromboembolism among patients with cancer.

## 2. Methods

### 2.1. Search Strategy

We based our systematic review and meta-analysis on the requirements of the Preferred Reporting Items for Systematic Reviews and Meta-Analysis (PRISMA) statement. The protocol was preregistered in the International Prospective Register of Systematic Reviews database (CRD42021252393). We searched the PubMed, Cochrane Library, and Scopus electronic databases on March 30, 2021 for studies published through February 2021. Following initial screening of the study title and abstract, we assessed candidate full-text articles for eligibility. Two researchers (F.U. and S.K.) then independently extracted data and determined whether the papers were candidates for full-text review. Disagreements were resolved by consensus with a third investigator or by the decision of the senior author (S.E.). Keywords in the search were “cancer” OR “carcinoma” OR “tumor” and “blood type” and “venous thrombosis” OR “venous thromboembolism” OR “pulmonary embolism”. Our primary outcome of interest was the development of venous thromboembolism.

### 2.2. Selection Criteria

Eligible studies were those that used univariate and multivariate logistic regression analysis in cohort studies to evaluate the association of ABO blood type with venous thromboembolism. Articles published in languages other than English, reviews, commentaries, and case series were excluded. If the same group published multiple articles using similar cohorts, we included either the most recent or the highest quality publication.

### 2.3. Data Extraction

Two authors (F.U. and S.K.) worked independently to extract the required data, including the first author’s name, country where patients were enrolled, number of patients, age, body mass index (BMI), cancer type, treatment, and ABO blood type. In cases where venous thrombosis developed, odds ratios (ORs) and 95% confidence intervals (CIs) were determined for ABO blood type. All discrepancies regarding data extraction were resolved by consensus.

### 2.4. Quality Assessment

After deciding which studies would be included, we used the Newcastle–Ottawa Scale to assess study quality [8,9], based on the Cochrane Handbook for systematic reviews. That scale uses a scale of from 0 to 9 to quantify three factors: Selection (1–4), Comparability (1–2), and Exposure (1–3). We identified the main confounders as important factors for the development of venous thromboembolism, and determined the presence of confounders by consensus and literature review. Studies with scores above 6 were considered “high-quality” choices.

## 3. Statistical Analysis

A forest plot was used to assess ORs from multivariate analyses of individual studies and to obtain a summary OR for the relationship between ABO blood type and the development of venous thromboembolism. Univariate logistic regression analyses were not included in the meta-analysis. We used the Cochrane Q test and I^2^ statistics to valuate heterogeneity in outcomes for the studies in this meta-analysis. Significant heterogeneity was indicated by *p* < 0.05 in the Cochrane Q test and ratio > 50% in I^2^ statistics, leading to the use of random effect models based on work by DerSimonian and Laird [10,11,12]. We used fixed-effect models to calculate pooled ORs for non-heterogeneous results and funnel plots to assess publication bias. All statistical analyses used Stata/MP 14.2 (Stata Corp., College Station, TX, USA). The level of statistical significance was set at *p* < 0.05.

## 4. Results

### 4.1. Study Selection

**Figure 1:**
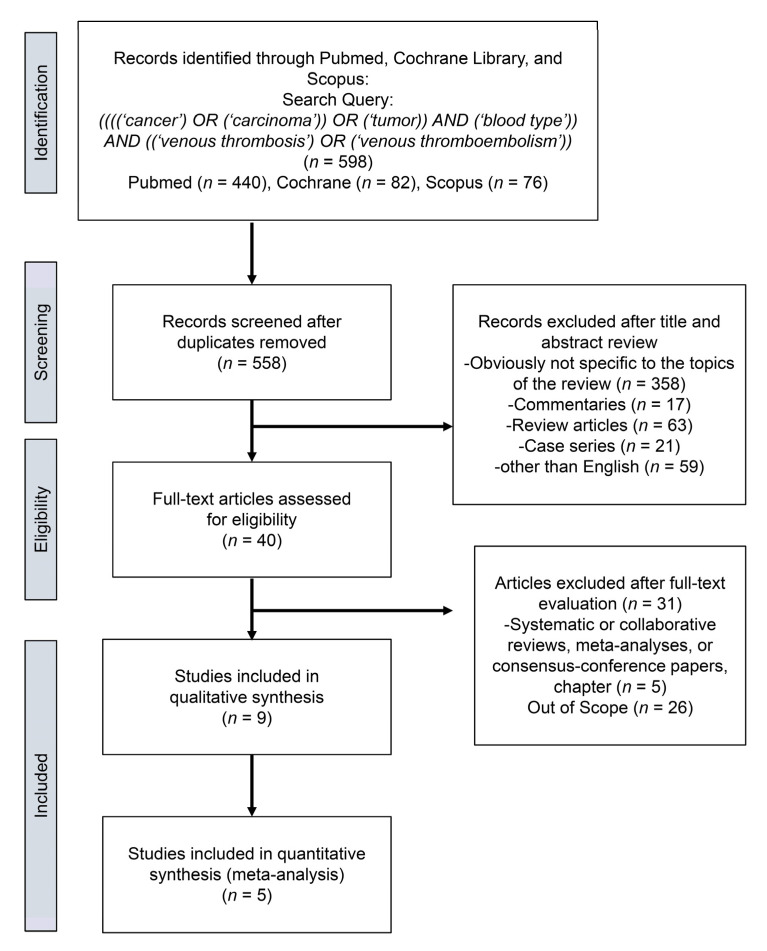
Preferred Reporting Items from the Systematic Review and Meta-Analysis (PRISMA) flow chart showing the process of article selection.

We found a total of 598 studies for initial assessment. From these, we removed 40 duplicates and excluded non-relevant studies, review articles, meeting abstracts, case reports, replies, editorials or commentaries, and studies in languages other than English. This left 40 studies for review, from which we identified nine studies for systematic review [13,14,15,16,17,18,19,20,21] and five studies [14,15,17,18,19] for qualitative meta-analysis (Figure 1).

### 4.2. Characteristics of the Included Studies

Table 1 lists the general characteristics of the eligible studies. All studies had a retrospective design and were published between 2004 and 2020. Patients were enrolled from Europe in 1 study, from North America in 7 studies, and from Asia in 1 study. Overall, 1118 of 25,884 patients developed venous thromboembolism. Table 2 and Table 3 list patient characteristics in the eligible studies. Various kinds of cancers were investigated, including glioma, prostate cancer, bladder cancer, acute lymphoblastic leukemia, pancreatic cancer, lymphoma, sarcoma, glioblastoma, tumors of the digestive system, lung cancer, breast cancer, and gynecological cancer. The treatment details were reported for seven studies in 24,996 patients: 24,473 of those patients (97.9%) underwent a surgical procedure (biopsy or surgical resection). The rate of development of venous thromboembolism in patients whose blood type was O and non-O was 9.2% and 13.6%, respectively. All of the eligible studies were retrospective, so pharmacologic prophylaxis was not standardized. In addition, the timing of diagnostic evaluation of patients for venous thromboembolism was based on symptom presentation and/or clinical evaluation, and most of the studies did not follow a precise protocol. Multivariate logistic regression analyses were not performed in three studies [13,16,20], and although Wang et al. evaluated the association of venous thromboembolism with ABO blood type, they reported a higher risk of each non-O blood type (A/B/AB) compared to O blood type (OR (95% CI); A: 2.072 (1.204–3.566), B: 1.944 (1.020–3.873), AB: 2.706 (1.432–5.114)). These four studies were excluded from our meta-analysis.

### 4.3. Meta-Analysis

The association of ABO blood type with development of venous thromboembolism in patients with cancers.

**Figure 2:**
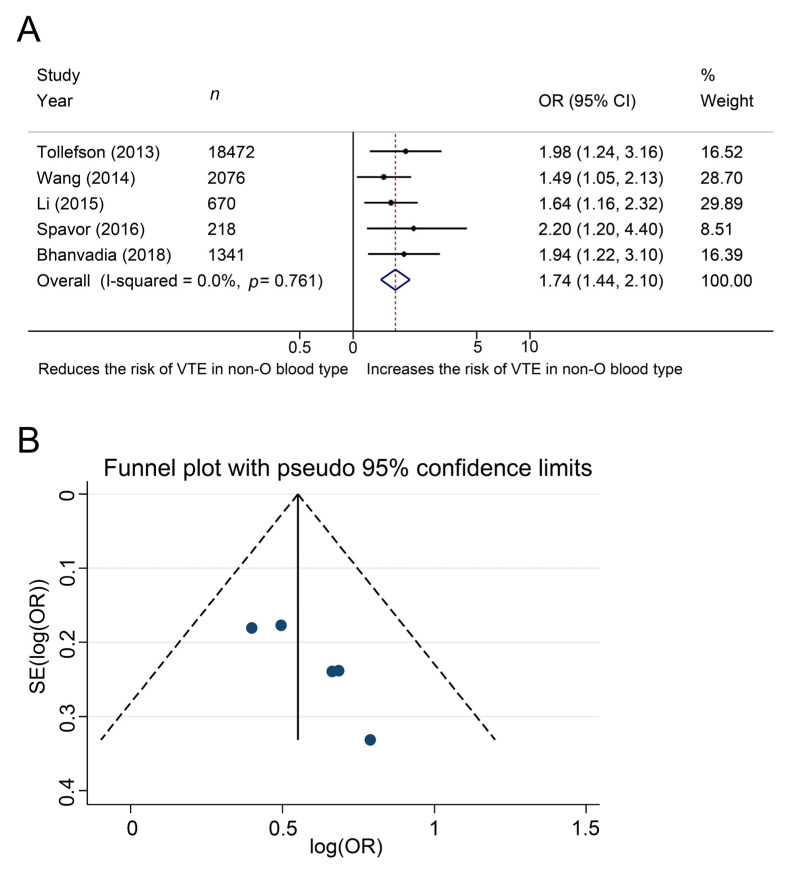
Forest (**A**) and funnel (**B**) plot showing the association of ABO blood type with development of venous thromboembolism among patients with cancer.

The impact of ABO blood type on development of venous thromboembolism was investigated in five studies, in a total of 22,777 patients with cancer. The Cochrane Q test (chi-square 1.86, *p* = 0.761) and the I^2^ test (I^2^ = 0.0%) showed no heterogeneity, so we used a fixed-effect model. The forest plots indicated a significant association between non-O blood type and the development of venous thromboembolism (pooled OR: 1.74, 95% CI, 1.44–2.10, z = 5.70, Figure 2A). The funnel plots showed no studies exceeding the pseudo 95% CI (Figure 2B).

### 4.4. Additional Analysis

The association of ABO blood type with the development of venous thromboembolism in patients after pelvic surgery for malignancy.

**Figure 3:**
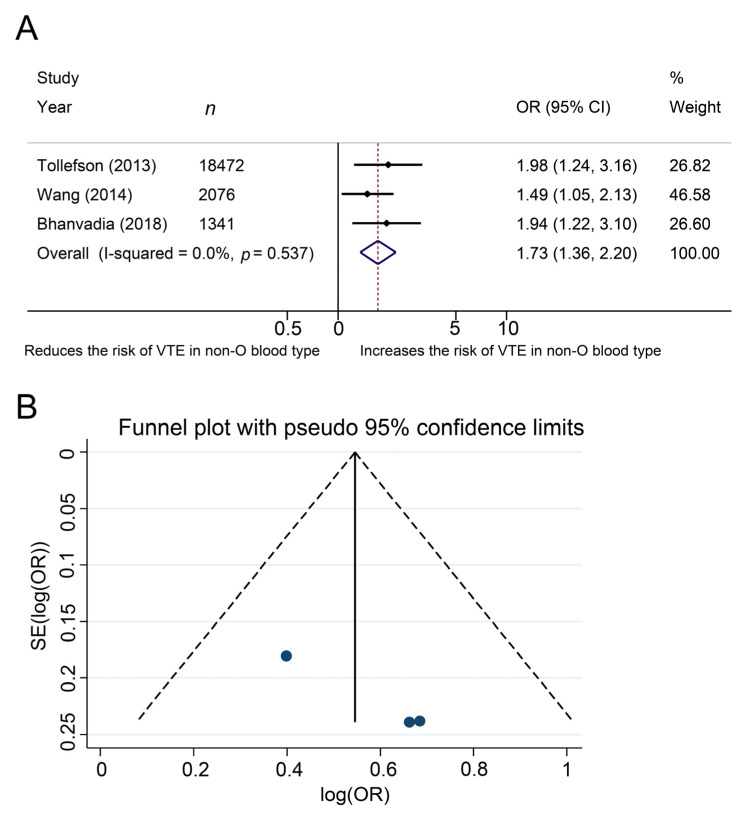
Forest (**A**) and funnel (**B**) plot showing the association of ABO blood type with development of venous thromboembolism among patients who underwent pelvic surgery for urological cancer.

The effects of ABO blood type on development of venous thromboembolism was investigated in three studies, in a total of 21,889 patients with cancer after pelvic surgery. The Cochrane Q test (chi-square 1.24, *p* = 0.537) and the I^2^ test (I^2^ = 0.0%) showed no heterogeneity, so we used a fixed-effect model. The forest plots demonstrated that non-O blood type was significantly associated with the development of venous thromboembolism (pooled OR: 1.73, 95% CI, 1.36–2.20, z = 4.43, Figure 3A). The funnel plots did not demonstrate any study over the pseudo 95% CI (Figure 3B).

## 5. Discussion

In this systematic review and meta-analysis, we analyzed five studies, enrolling a total of 22,777 patients with various kinds of cancers. To our knowledge, this is the first meta-analysis to elucidate the impact of ABO blood type on venous thromboembolism among patients with cancer. In this study, a non-O blood type was significantly associated with the development of venous thromboembolism in patients with cancer. Additionally, in subgroup analysis, we reviewed the impact of ABO blood type on the development of venous thromboembolism in 21,889 patients who underwent pelvic surgery for urological cancers, with the same results as in our main analysis.

The protective role for O blood type may be explained in part by the differential survival of circulating von Willebrand factor (vWF) and clotting factor VIII (FVIII) for O blood type compared with non-O blood type. VWF has been shown to stabilize FVIII and to prevent its proteolytic degradation [22], and plasma concentrations of vWF and FVIII are approximately 25% higher in patients with non-O blood type than in those with O blood type [23,24]. Our findings, that cancer patients with non-O blood type are more likely to experience venous thromboembolism, are consistent with previous reports in the hematologic literature. El-Galary et al. reported a prospective study that non-O blood type is significantly associated with the risk of venous thromboembolism in Danish people with no previous diagnosis of cancer [25]. In addition, Nauffal et al. recently evaluated the association of ABO blood types with cardiovascular events in coronavirus disease 2019 (COVID-19), which can also cause venous thromboembolism. Those findings showed that non-O (A) blood type COVID-19 patients tended to be more at risk of thrombotic events than those patients with O blood type [26].

Venous thromboembolism is a serious and frequent complication of pelvic surgery for malignancy. Prostatectomy and cystectomy have been reported to be associated with increased risk of venous thromboembolism [19,27], and the American Urological Association has recommended considering thromboprophylaxis in patients scheduled for urological surgery [28]. However, venous thromboembolism after pelvic surgery is infrequent, and pharmacological prophylaxis may be associated with increased postoperative complications such as bleeding and lymphocele formation [29,30], so this prophylaxis remains underused in patients treated with pelvic surgery for malignancy. Although further prospective cohort studies are needed, the results suggest that considering ABO blood type in refining the risk assessment of post-operative venous thromboembolism in urological cancer patients may be useful to guide decisions on venous thromboembolism prophylaxis.

The present study represents the first systematic review and meta-analysis to assess the impact of ABO blood type on development of venous thromboembolism among patients with cancer. The study has some limitations. All the included studies are retrospective and may have been affected by selection bias, which may have been increased by our rejection of articles published in languages other than English. In addition, patients with various kinds of cancer were included in our meta-analysis. However, more than 90% of patients were post-operative urological cancer patients, which could be a limitation to the generalizability of the results. Thus, further studies are needed to verify these results in each kind of cancer. As these were retrospective studies, the identification of venous thromboembolism was not strictly defined in each study, but instead was based on the patient’s reporting of symptoms and the physicians’ judgment regarding suspected venous thromboembolism. Prospective cohort studies will be required to address this problem. Furthermore, pharmacologic prophylaxis was not uniformly utilized across the studies. However, patients were treated similarly at every given timepoint during each study, irrespective of blood type. Thus, the differences in pharmacologic prophylaxis should not affect the results of each study.

## 6. Conclusions

Our systematic review and meta-analysis show that non-O blood type is associated with higher risk of the development of venous thromboembolism among patients with cancer. Blood type is routinely determined preoperatively by objective and standardized methods, and our results suggest that these blood type results are useful for risk stratification and potentially for encouraging appropriate strategies for implementation of the prophylactic treatment strategy in venous thromboembolism management, especially after pelvic surgery for urological cancers.

## Figures and Tables

**Table 1:** Characteristics of all articles included in the study.

					VTE		
First Author of Study and [Ref.]	Country	Recruitment Period	Study Design	Total	Yes	No	NOS
Streiff et al. [13]	USA	1991–2001	Cohort, retrospective	130	28	102	6
Tollefson et al. [14]	USA	1987–2010	Cohort, retrospective	18472	271	18201	7
Wang J et al. [15]	USA	1980–2005	Cohort, retrospective	2076	216	2060	7
Mizrahi et al. [16]	Canada	1995–2013	Cohort, retrospective	523	56	467	6
Li et al. [17]	USA	NR	Cohort, retrospective	670	236	434	6
Spavor et al. [18]	Canada	NR	Cohort, retrospective	218	63	155	6
Bhanvadia et al. [19]	USA	2003–2015	Cohort, retrospective	1341	90	1251	7
Heenkenda et al. [20]	Sweden	NR	Cohort, retrospective	139	47	92	6
Wang G et al. [21]	China	2018–2019	Cohort, retrospective	2315	131	2174	7

NOS, Newcastle-Ottawa Scale; VTE, venous thromboembolism; NR, not reported.

**Table 2:** Comparison of characteristics of patients between VTE and non VTE.

		Age (y)			Gender (Male)			Blood Type			BMI		
First Author of Study and [Ref.]	No. of pts	Total	No VTE	VTE	Total	No VTE	VTE	Total	No VTE	VTE	Total	No VTE	VTE
Streiff et al. [13]	130	median 55 (47–66)	median 56 (44–66)	median 54 (51–62)	83	64 (63%)	19 (68%)	NR	NR	NR	median 25.8 (24–28.3)	median 26 (24–28)	median 26 (25–29)
Tollefson et al. [14]	18,472	median 63 (58–68)	median 63 (58–68)	median 65 (59–68)	18,472	18,201 (100%)	271 (100%)	NR	NR	NR	median 27.7 (25.4–30.3)	median 27.7 (25.4–30.3)	median 27.8 (25.7–30.5)
Wang J.K. et al. [15]	2076	NR	median 68 (62–74)	median 69 (61–76)	1670	1498 (80.5%)	172 (79.6%)	O: 865 (41.7%) Non-O: 1143 (55.1%) Missing: 61 (3.3%)	O: 794 (44.1%) Non-O: 1007 (55.9%) Missing: 59 (3.2%)	O: 71 (34.3%) Non-O: 136 (65.7%) Missing: 9 (4.2%)	NR	≥30: 437 (23.6%) <30: 1515 (76.4%) Missing: 8 (0.4%)	≥30: 71 (32.8%) <30: 145 (67.1%) Missing: 0 (0.0%)
Mizrahi et al. [16]	523	mean 6.5 SD 4.4	≥10 y: 101 (20.4%) <10 y: 395 (79.6%)	≥10 y: 20 (35.7%) <10 y: 36 (64.3%)	302	270 (57.8%)	32 (57.1%)	O: 221 (42.3%) A: 230 (44.0%) AB: 11 (2.61%) B: 60 (11.5%)	O: 207 (44.7%) Non-O: 259 (55.3%)	O: 14 (25%) Non-O: 42 (75%)	NR	NR	NR
Li et al. [17]	670	median 62 (31-87)	≤60 y: 193 (44.5%) >60 y: 241 (55.5%)	≤60 y: 98 (41.5%) >60 y: 138 (58.5%)	388	249 (57.4%)	139 (58.9%)	O: 251 (37.5%) A: 328 (49.0%) AB: 19 (2.8%) B: 72 (10.7%)	O: 178 (41.0%) A: 200 (46.1%) AB: 9 (2.1%) B: 47 (10.8%)	O: 73 (30.9%) A: 128 (54.2%) AB: 10 (4.2%) B: 25 (10.6%)	NR	>30: 130 (30.2%)	>30: 88 (37.6%)
Spavor et al. [18]	218	NR	mean SD 8.5 ± 5.4	mean SD 7.1 ± 4.6	121	82 (53%)	39 (62.3%)	NR	NR	NR	NR	NR	NR
Bhanvadia et al. [19]	1341	median 70	NR	NR	NR	NR	NR	O: 595 (44.4%) A: 520 (38.8%) AB: 63 (4.7%) B: 163 (12.2%)	NR	NR	27	NR	NR
Heenkenda et al. [20]	139	NR	median 60 (25–76)	median 58 (39–69)	NR	58 (63%)	29 (62%)	O: 49 (35.3%) A: 67 (48.2%) AB: 5 (3.6%) B: 18 (12.9%)	O: 38 (41%) A: 45 (49%) AB: 3 (3%) B: 6 (7%)	O: 11 (23%) A: 22 (47%) AB: 2 (4%) B: 12 (26%)	NR	NR	NR
Wang G et al. [21]	2315	mean 52 (18–89)	<65 y: 1781 (81.5%) ≥65 y: 403 (18.5%)	<65 y: 111 (84.7%) ≥65 y: 20 (15.3%)	1084	1025 (46.9%)	59 (45%)	O: 677 (29.2%) A: 619 (26.7%) AB: 245 (10.6%) B: 774 (33.4%)	O: 656 (30.0%) A: 577 (26.4%) AB: 225 (10.3%) B: 726 (33.2%)	O: 21 (16.0%) A: 42 (32.1%) AB: 20 (15.3%) B: 48 (36.6%)	NR	≥30: 814 (37.3%)	≥30: 58 (44.3%)

BMI, body mass index; No, number; pts, patients; NR, not reported; VTE, venous thromboembolism.

**Table 3:** Clinical characteristics of study cohorts.

First Author of Study and [Ref.]	Cancer Type	Treatment Content	Pharmacoprophylaxis	VTE Criteria
Streiff et al. [13]	Glioma	Surgery ± local or systemic chemotherapy	Some patients (21%) received subcutaneous heparin	DVT: Duplex ultrasonography or venography. PE: High-probability ventilation perfusion scan, a positive spiral CT, or pulmonary angiography
Tollefson et al. [14]	Prostate cancer	Radical prostatectomy	Not standardized	NR
Wang J.K. et al. [15]	Bladder cancer	Radical cystectomy	Not standardized	DVT: Duplex ultrasonography or venography. PE: Arteriography, ventilation/perfusion scan, or enhanced CT. With at least one sign of VTE
Mizrahi et al. [16]	Acute lymphoblastic leukemia	Intrathecal chemotherapy	None	Doppler ultrasound, CT scan, MRI, cardiac ultrasound, or ventilation/perfusion lung scan
Li et al. [17]	Pancreatic cancer	NR	None	Radiological imaging
Spavor et al. [18]	Acute lymphoblastic leukemia: 92 (42.2%) Lymphoma: 45 (20.6%) Sarcoma: 26 (11.9%) Others: 55 (25.2%)	NR	NR	Ultrasonography, CT scan, MRI, venography, ventilation perfusion scan or echocardiography with at least one sign of VTE
Bhanvadia et al. [19]	Bladder cancer	Radical cystectomy	Immediate postoperative Coumadin Or Postoperative subcutaneous heparin	Ultrasonography, ventilation/perfusion scan or pulmonary angiography with at least one sign of VTE
Heenkenda et al. [20]	Glioblastoma	Surgery/biopsy + RT + temzolomide	Pre- and post- postoperative tinzaparin	Ultrasound sonography or pulmonary angiography with at least one sign of VTE
Wang G et al. [21]	Lymphoma: 1000 (43.2%) Lung tumor: 46 (2.0%) Tumor of digestive system: 532 (23.0%) Urologic tumor: 72 (3.1%) Breast tumor: 531 (22.9%) Gynecological tumor: 42 (0.043%) Others: 91 (3.9%)	Peripherally inserted central catheter	None	Doppler ultrasound with at least one sign of VTE

CT, computed tomography; DVT, deep vein thrombosis; MRI, magnetic resonance imaging; PE, pulmonary embolism; NR, not reported; VTE, venous thromboembolism.

## Data Availability

All data generated and analyzed during this study are included as part of this study.
